# Save the Children

**Published:** 1920-02-21

**Authors:** 


THE PUBLIC WELL-BEING—(*continued*).

### Save the Children.

### Appeal for those in Famine Areas.

We have received, and gladly draw attention to, the appeal of the Labour Committee of the Save the Children Fund. It puts the stark facts of the case succinctly and moderately, and though the appeal is necessarily a sectional appeal, the facts are common ground which should be present to the minds of all. They are contemptible people who at any time allow the passions of war to be exercised on little children, and it should be the privilege of all humane people to do what is in their power to relieve the children from the consequences of war. Those who in their narrowness criticised that plain axiom of human feeling, did so merely from lack of imagination. They held that the children of enemies should not be succoured, but if a suffering child of the enemy had stood before them they would have been moved to help if they were not mere brutes of the basest type. Criticism, however, is now virtually dead. Nurseries are sacred ground, and Francis Thompson expressed what is in all feeling hearts when he wrote to a child thought to be dying, "Look for me in the nurseries of Heaven."

The Labour appeal says the position in the famine areas of Europe is so serious that Mr. Hoover has recently stated that approximately three and a-half million children will perish during the coming winter unless relief is sent to them. But it is not only the death-rate that must be considered; the children who live will grow up diseased, or weakened by long under-nourishment, and half-starved bodies must inevitably mean half-starved minds. They cannot possibly grow into healthy or active citizens, and if Europe is peopled by a generation that is physically and mentally warped, there is little hope of progress or of establishing a sound international relationship.

The labour organisations in neutral countries such as Sweden and Switzerland have already done much by taking in children from the famine areas, feeding and housing them. Although this cannot be done by this country, there are many ways in which we can help, and they feel sure that the workers here will not fail to respond to the cry of the children of their comrades abroad.

The position in the coming winter even in Great Britain will be difficult, with the high prices of milk and other foods, but whatever difficulties have to be faced here are not to be compared with the sufferings which thousands of children are undergoing in the distressed countries. If all the members of each labour organisation would give something, however small the sum, the result would be to save not merely the health but actually the lives of great numbers of suffering children.

As a matter of fact, an attempt is being made by a newly-formed Famine Area Children's Hospitality Committee in London which hopes to arrange for hospitality in this country for 5,000 children in addition to the relief that is given on the spot. It is a great and beneficent undertaking.

